# A Dramatic Response of a Thyroid Lymphoma to R-CHOP Chemotherapy Reversing Mechanical Airway Obstruction and Respiratory Failure

**DOI:** 10.1155/2022/3719320

**Published:** 2022-06-27

**Authors:** Zeinab Alnahas, Mohamad H. Horani

**Affiliations:** ^1^Department of Internal Medicine, Cairo University, Giza, Egypt; ^2^Chandler Regional Medical Center, AZ 1955 W Frye Rd, Chandler, AZ 85224, USA

## Abstract

Primary thyroid lymphoma is an extremely rare thyroid malignancy that usually occurs in patients with preexisting Hashimoto thyroiditis and commonly presents in older women. The most common type is non-Hodgkin's lymphoma of B-cell origin, particularly diffuse large B-cell lymphoma (DLBCL), which is primarily treated with chemotherapy and radiotherapy. We reported an 83-year-old woman with a past medical history of hypothyroidism who suffered dysphagia and dyspnea secondary to a large thyroid mass. Her CT neck scan showed an enlarged thyroid mass with pathological cervical lymphadenopathy and marked tracheal narrowing. The ultrasound-guided biopsy confirmed the diagnosis of DLBCL of the thyroid. A few days later, she experienced respiratory distress and failure that required endotracheal intubation and mechanical ventilation. She was not considered for tracheostomy or surgical interventions, and after discussion with her family, the decision was made to start R-CHOP therapy (rituximab, cyclophosphamide, doxorubicin, vincristine, and prednisone) which resulted in a marked reduction of the thyroid size and reversal of the mechanical airway obstruction, enabling her extubation. This case report demonstrated the dramatic response of a large thyroid lymphoma to R-CHOP therapy, reducing the thyroid size and its fatal obstructive complications, including mechanical airway obstruction, within a few days of the initiation of R-CHOP therapy.

## 1. Background

Primary thyroid lymphoma is extremely rare and accounts for <5% of all thyroid malignancies and <2% of extranodal lymphomas, with an estimated incidence of 2 per 1 million each year [1]. It usually occurs in patients with preexisting Hashimoto thyroiditis who are at 67% greater risk of developing thyroid lymphoma compared to those without thyroiditis. Primary thyroid lymphoma commonly affects women, typically between 60 and 70 years [2]. According to the 2008 World Health Organization's classification of lymphomas, almost all thyroid lymphomas are non-Hodgkin's lymphoma of B-cell origin, with diffuse large B-cell lymphoma (DLBCL) being the most common subtype accounting for 50–70%, followed by mucosa-associated lymphoid tissue (MALT) lymphoma, representing 25–30% of all thyroid lymphomas [3]. DLBCL tends to follow an aggressive course and usually presents with an enlarging anterior neck mass that might cause local obstructive symptoms in up to 30% of the patients, including dyspnea, dysphagia, hoarseness, and stridor [4]. However, B-symptoms such as fever, weight loss, and night sweats are uncommon and present in about 10% of the patients [5]. Thyroid lymphoma treatment and prognosis depend on the tumor's histology and staging. However, surgery has a limited role, and the standard treatment for thyroid lymphoma is chemotherapy and radiotherapy [6]. This case report shows a dramatic response to chemotherapy in a patient with mechanical airway obstruction due to large thyroid lymphoma, which facilitated her extubation.

## 2. Case Presentation

**Figure 1:**
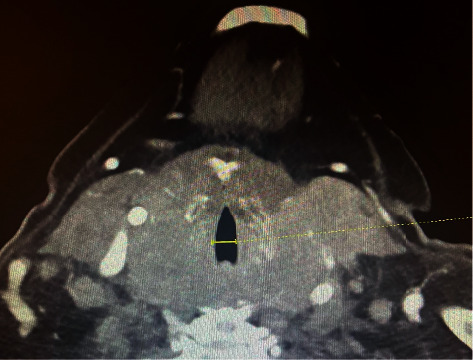
CT neck scan with contrast showed a markedly enlarged bilateral thyroid gland measured approximately 7.8 × 7.2 cm, with marked transverse narrowing of the trachea at the level of the thyroid gland to approximately 8.6 mm (solid yellow line).

**Figure 2:**
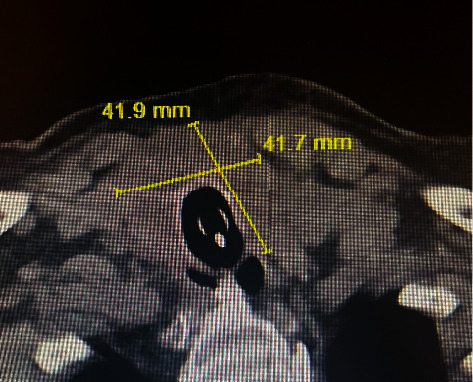
Following chemotherapy, the patient's CT neck scan with contrast showed a significant decrease in the thyroid size measured approximately 4.2 × 4.2 cm, with marked improvement in tracheal narrowing.

An 83-year-old woman with a past medical history significant for type 2 diabetes, coronary heart disease, and hypothyroidism controlled on L-thyroxine replacement therapy was presented with 3 months history of gradually progressive dysphagia. It was associated with left-sided neck pain with swallowing, choking on food, and shortness of breath. She denied fever, chills, or excessive sweats. Local examination of her neck revealed a large firm thyroid mass associated with left cervical lymphadenopathy. Her CT neck scan with intravenous contrast showed an enlarged, probably malignant thyroid gland with a marked amount of adjacent pathologic lymph nodes and marked transverse narrowing of the trachea to approximately 8.6 mm with no retrosternal extension (Figure 1). Also, her CT abdominal scan with intravenous contrast showed a 3.6 cm lesion of the medial right hepatic lobe (segment 8) concerning for metastases. The patient underwent an ultrasound-guided thyroid biopsy, and the results confirmed the diagnosis of DLBCL of the neck and thyroid. A few days later, the patient experienced respiratory distress and failure secondary to mechanical airway obstruction that required urgent intubation and mechanical ventilation. The discussion among different specialties, including general surgery, concluded that the patient was not a candidate for surgical intervention or tracheostomy, and recommendations were to proceed to palliative chemotherapy. She started R-CHOP chemotherapy (rituximab, cyclophosphamide, doxorubicin, vincristine, and prednisone), followed by a marked reduction in the thyroid size and relieving of the tracheal compression on the following CT neck scan (Figure 2). Her condition dramatically improved, and she was extubated after 5 days of receiving the first cycle of R-CHOP therapy.

## 3. Discussion

Although the development of primary thyroid lymphoma on the top of preexisting thyroiditis is uncommon and usually occurs in 0.5% of cases, most lymphoma cases arise in a background of thyroiditis. Thyroid lymphoma should be suspected in patients with thyroiditis experiencing a rapidly enlarging neck mass [7]. Ultrasound-guided needle biopsy of the thyroid mass is the standard diagnostic testing, although in some cases, open biopsy is needed to confirm the diagnosis [1]. Pretreatment evaluation using comprehensive imaging of the chest, abdomen, and pelvis is crucial for determining the tumor staging, predicting prognosis, and establishing optimal therapeutic options [4]. Traditionally, the primary treatment for thyroid lymphomas was surgery and radiotherapy. However, due to significant relapse rate, poor survival rates, and the tumor sensitivity to chemotherapy and radiotherapy, the current standard practice, especially for DLBCL, is multimodality therapy with combination chemotherapy and loco-regional radiotherapy [8].

Multiagent chemotherapy generally includes cyclophosphamide, doxorubicin, vincristine, and prednisone (CHOP), and recently, the monoclonal anti-CD20 rituximab was added to the CHOP regimen (R-CHOP) [9]. Rituximab can act synergistically with chemotherapy and induce cell lysis of thyroid lymphoma through direct and indirect mechanisms, including induction of apoptosis, complement-mediated cytolysis, and antibody-dependent cell cytotoxicity [10]. Treatment of thyroid lymphoma with R-CHOP significantly improved the complete response rates and reduced treatment failure and relapse rates. Moreover, it increased the overall survival rates compared with CHOP alone without significant additional toxicity. In a small case series by Jonak et al. [11], who studied the effectiveness of R-CHOP therapy in elderly patients with DLBCL of the thyroid who were considered unfit for surgery or inoperable, treatment with R-CHOP resulted in complete remission of the lymphoma without evidence of disease recurrence for 2 years after initiation of the therapy [11]. In our case report, initiation of R-CHOP therapy in an elderly patient with a large thyroid lymphoma complicated by mechanical airway obstruction and respiratory failure was associated with a marked reduction in the thyroid size and reversal of the mechanical airway obstruction within a few days of receiving the first cycle of R-CHOP therapy.

## 4. Conclusion

Our case report demonstrates dramatic reversal of mechanical airway obstruction caused by a thyroid lymphoma within a few days of initiation of R-CHOP therapy. These cases are usually considered terminal and do not undergo aggressive surgical interventions. R-CHOP chemotherapy needs to be considered in these cases before considering hospice care.
